# A Simple and Efficient Solar Interfacial Evaporation Device Based on Carbonized Cattail and Agarose Hydrogel for Water Evaporation and Purification

**DOI:** 10.3390/membranes12111076

**Published:** 2022-10-30

**Authors:** Liang Wang, Jilei Wei, Chen Zhou, Shengyang Yang

**Affiliations:** 1 School of Chemistry and Materials Engineering, Nanjing Polytechnic Institute, 188 Xinle Road, Nanjing 210048, China; 2 Department of Chemistry and Chemical Engineering, Yangzhou University, 180 Siwangting Road, Yangzhou 225002, China; 3 Department of Physical Sciences, University of Central Missouri, Warrensburg, MO 64093, USA

**Keywords:** solar water evaporation, seawater desalination, wastewater purification, carbonized cattail, hydrogel

## Abstract

One of the main trends in the development of solar interface evaporation technology is the simple, efficient, and environmentally friendly bio-based evaporation device. However, the development of bio-based evaporators with high water evaporation rates and good pollution removal capability is a significant challenge. Here, we present a carbonized cattail–agarose hydrogel (CCAH) membrane with numerous microchannels resembling bamboo knots, exceptional hydrophilicity, outstanding light absorption capability, and potent adsorption. Under one solar irradiation, its evaporation rate and efficiency reached 1.93 kg m^−2^ h^−1^ and 95.8%, respectively. More importantly, the CCAH membrane produces steam water that is almost totally free of salts (Na^+^, K^+^, Mg^2+^, and Ca^2+^), heavy metal ions (Pb^2+^, Cd^2+^, and Cr^2+^), and organic dyes (Rhodamine B, methylene blue, and methyl orange). The CCAH membrane is highly promising for the use of saltwater desalination and wastewater recovery to help people in impoverished areas with water scarcity problems.

## 1. Introduction

One of the most pressing current issues is the global water crisis, which is a barrier to the advancement of society [1,2,3]. Wastewater recovery and seawater desalination are two practical solutions to the water crisis [4,5]. For wastewater recovery, the conventional water treatment method is typically combined with membrane filtration, while distillation or reverse osmosis membrane filtration is typically utilized for seawater desalination [6,7]. However, these advanced water treatment technologies are not appropriate for economically underdeveloped coastal or isolated areas because of their high investment and operating costs [8,9,10,11]. The use of renewable energy (wind, solar, biomass, and carbon dioxide) for power generation, fuel manufacturing, and water treatment can lower global energy consumption and have a substantial positive influence on the economy [12,13,14,15]. Among these, the rapidly developing solar interface evaporation technology, which has a low operating cost and is used for wastewater treatment and saltwater desalination, may offer a solution to the issue of water scarcity in underdeveloped and remote areas [1,3]. The cutting-edge research areas for solar evaporators are the functional materials and bio-inspired structure design. Researchers have chosen certain common functional materials for the purpose of water purification, including carbon nanotube [16], graphene [17,18,19,20], metal [21,22,23,24], biochar [25,26], pyrrole [27,28], melamine polymer [29,30], porous alumina and silica [31,32], and hydrogel [33,34,35,36,37,38]. The creation of a solar evaporator with exceptional photothermal conversion performance and high evaporation efficiency is possible through the combination and structural optimization of various functional materials [39,40]. Although these devices have been developed for almost a decade, numerous issues still prevent their widespread applications, including expensive raw material costs, poor anti-fouling and salt resistance, and inadequate endurance in harsh settings [41,42,43]. 

Nature has provided inspiration for some of the most effective solar interface evaporation devices. Biomaterials such as wood [44], mushrooms [45], loofah [46], bamboo [47], and Enteromorpha prolifera [48] were employed for solar interfacial evaporation after being carbonized. Great light absorption, strong adsorption, and complex micro-pipeline structures are typical properties of low-cost biochar materials [25,26,44,45,46,47,48]. However, it is challenging to develop an evaporation device that can efficiently evaporate water as well as remove the majority of the salts and other contaminants present in raw water, since bio-based materials often have inherent limitations in mechanical performance. Therefore, hybrid composites are usually utilized to improve the strength property of the membranes for long-term use in water purification.

Cattail is a prominent aquatic commercial plant in China with a high biological yield and low raw material cost. Raw cattail is mainly composed of a large number of fibers, which have a unique microstructure similar to bamboo knot. Researchers have successfully removed phenol from an aqueous solution using activated carbon made from cattail as an adsorbent [49]. In this study, we successfully created a simple solar interface evaporation device using carbonized cattail and agarose hydrogel. Due to the abundance of bamboo-like micron channels, strong light absorption capabilities, and superior hydrophilicity of the carbonized cattail, the device is capable of transporting and evaporating water as well as performing an efficient photothermal conversion. Moreover, it has high rates of salt, heavy metal ion, and organic dye removal in photothermal water purification. Carbonized cattail and agarose hydrogel are harmless to the environment and the human body. Thus, a simple, efficient, low-cost, and environmentally friendly solar interface evaporation device based on carbonized cattail and agarose hydrogel (CCAH) is presented for the first time in this study. The application of this device in real-world wastewater recovery and seawater desalination is highly promising. 

## 2. Experimental Section

### 2.1. Materials

Cattail was picked from a riverside by ourselves in Jining City, China. Lithium hydroxide monohydrate (LiOH·H_2_O, 99.98%), urea (CH_4_N_2_O, 99.5%), agarose (C_24_H_38_O_19_, for biochemistry), and magnesium sulfate anhydrous (MgSO_4_, AR) were purchased from Aladdin Chemical Reagent Co., Ltd. (Shanghai, China) Ethanol (C_2_H_6_O, 99.7%), methyl orange (C_14_H_14_N_3_NaO_3_S, 96%), methylene blue (C_16_H_18_N_3_SCl, AR), Rhodamine B (C_28_H_31_ClN_2_O_3_, 99.0%), lead (II) acetate trihydrate (Pb(CH_3_COO)_2_·3H_2_O, 99.99%), chromium acetate (Cr(CH_3_COO)_3_, 99.9%), and cadmium acetate dihydrate (Cd(CH_3_COO)_2_·2H_2_O, 99.99%) were purchased from Shanghai Macklin Biochemical Technology Co., Ltd. (Shanghai, China) Sodium chloride (NaCl, AR), potassium chloride (KCl, AR), and calcium chloride anhydrous (CaCl_2_, AR) were purchased from Sinopharm Chemical Reagent Co., Ltd. (Shanghai, China) Ultrapure water with an electrical resistivity of 18.2 MΩ.cm was utilized as the experiment water. 

### 2.2. Preparation of Agarose Hydrogel 

A total of 7 g of lithium hydroxide monohydrate (LiOH·H_2_O) and 16 g of urea (CH_4_N_2_O) were completely dissolved in an appropriate amount of water, and then the above mixture was transferred to a 100 mL volumetric flask for constant volume operation [50]. A total of 10 mL of freshly prepared lithium hydroxide-urea solution taken out of the volumetric flask was combined with 0.15 g agarose and carefully stirred until dissolved. The mixture was then frozen for 4 h at −20 °C in a refrigerator. Next, it was fully stirred during the thawing process. Finally, the mixture was washed by centrifugation (desktop high speed centrifuge: H1850, Hunan Xiangyi Laboratory Instrument Development Co., Ltd., Changsha, China) to obtain agarose hydrogel for further use. 

### 2.3. Preparation of Carbonized Cattail–Agarose Hydrogel (CCAH) Membrane 

**Figure 1:**
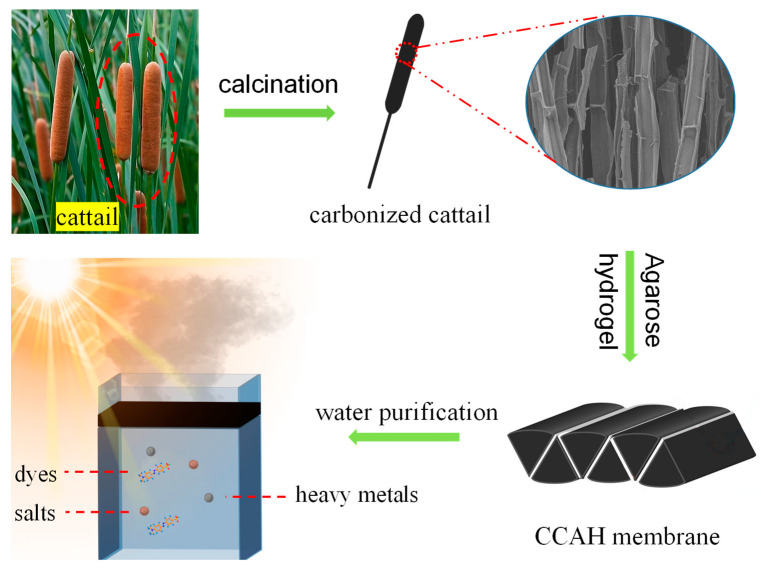
Schematic diagram of preparation process and application of carbonized cattail–agarose hydrogel (CCAH) composite membrane for solar water evaporation and water purification.

The cleaned and dried cattail was first calcined for 2 h at 500 °C in a nitrogen environment with a temperature increase rate of 5 °C min^−1^ (tube furnace: OTF-1200X-S, Hefei Kejing Material Technology Co., Ltd., Hefei, China). After naturally cooling to room temperature, the carbonized cattail (CC) was cut into six identical, 30-mm-long triangular columns. Then, the previously prepared glue (agarose hydrogel) was evenly applied to the sides of the triangular column. Finally, a carbonized cattail–agarose hydrogel (CCAH) membrane was prepared by combining six identical triangular columns into a cuboid by pasting and trimming (Figure 1).

### 2.4. Solar-Driven Water Evaporation

Solar-driven water evaporation, wastewater purification, and seawater desalination experiments were carried out in a square quartz cup with an inner side length of 30 mm. Under the action of simulated sunlight (1 kW m^−2^, CEL-HXF300, Zhongjiao Jinyuan Technology Co., Ltd., Beijing, China) in the presence of an AM 1.5 G filter, the CCAH membrane self-floated on the pure water surface in the quartz cup to conduct the water evaporation experiment. The evaporation process was performed in a hermetic room with a temperature of ~28 °C and humidity of ~35%. By weighing the remaining water using an electronic balance (LE204E, Mettler-toledo Apparatus Co., Ltd., Shanghai, China, Accuracy: 0.1 mg), the rate of water evaporation was calculated.

### 2.5. Solar-Driven Wastewater Purification and Seawater Desalination

The wastewater purification process was the same as the above process, apart from the involvement of contaminants and a closed transparent quartz collector. In order to simulate wastewater, organic dyes and heavy metals were used in this work. To be more precise, the dye wastewater was prepared using pure water and a type of dye (methyl orange, methylene blue, or Rhodamine B), at a concentration of 10 mg L^−1^. Two concentrations of heavy metal wastewater (Cr^2+^, Cd^2+^, or Pb^2+^) were produced, 10 mg L^−1^ and 100 mg L^−1^, respectively. Following solar-driven evaporation of wastewater containing heavy metal ions or organic dyes, the remaining pollutants were then determined by analyzing the steam condensation water produced by the unit. The procedure for desalinating seawater is similar to that for purifying wastewater, with the exception that four salts (Na^+^, K^+^, Mg^2+^, and Ca^2+^) are employed in place of pollutants. The concentrations of four ions (Na^+^, Mg^2+^, K^+^, and Ca^2+^) in the simulated seawater were 10,780 mg L^−1^, 1298 mg L^−1^, 400 mg L^−1^, and 410 mg L^−1^, respectively. 

### 2.6. Characterization

Scanning electron microscopy (SEM) images of raw cattail and carbonized cattail (CC) were obtained with a field-emission scanning electron microscope system (GeminiSEM 300, Carl Zeiss AG, Oberkochen, Germany). The Fourier transform infrared spectra (FT-IR) of raw cattail and CC were obtained using KBr pellets on a Bruker spectrometer (Tensor 27, Bruker Optics GmbH, Ettlingen, Germany) from 400 to 4000 cm^−1^. X-ray photoelectron spectroscopy (XPS) of CC was performed with an electron spectrometer (ESCALAB 250Xi, Thermo Fisher Scientific, Waltham, MA, USA). UV–vis spectra and Solid UV–vis diffuse reflectance spectra of raw cattail and CCAH membrane were obtained using a UV-Vis spectrophotometer (i5, Hanon Instruments Co., Ltd., Jinan, China) and a UV-Vis-NIR spectrophotometer (Cary-5000, Varian, Inc., Palo Alto, CA, USA), respectively. The contact angles of raw cattail and CCAH membrane were measured with a video contact angle measuring instrument (OCA20). Time-dependent digital infrared thermal images of pure water with CCAH membrane were observed by a thermal infrared imager (testo 869, testo). The concentrations of ions in the stock solution and evaporated water were detected by taking an inductively coupled plasma emission spectrometer (ICP-OES, Optima 8300, PerkinElmer, Waltham, MA, USA). The concentrations of dyes in the stock solution and evaporated water were determined by a high-performance liquid chromatography (HPLC, Agilent 1260, Agilent Technologies, Santa Clara, CA, USA) and an ultraviolet-visible spectrophotometer (i5, Hanon Instruments Co., Ltd., Jinan, China).

### 2.7. Calculation of Evaporation Efficiency

The evaporation efficiency (η) is calculated by the formula [36]:

η = q_m_(C_p_×ΔT + h_LV_)/P_i_

in which q_m_ is the evaporation rate under solar illumination (kg m^−2^ h^−1^), C_p_ is the specific heat capacity of water and a constant of ~4.18 kJ kg^−1^ K^−1^, ΔT indicates the increased temperature of the absorber surface (K), h_LV_ is the actual enthalpy of water vaporization (kJ kg^−1^, note: the latent heat of water evaporation in the CCAH membrane was estimated by the evaporation experiments with and without the CCAH membrane under dark conditions at room temperature), and P_i_ is the power density of solar illumination on the CCAH membrane (1 kW m^−2^).

## 3. Results and Discussion 

**Figure 2:**
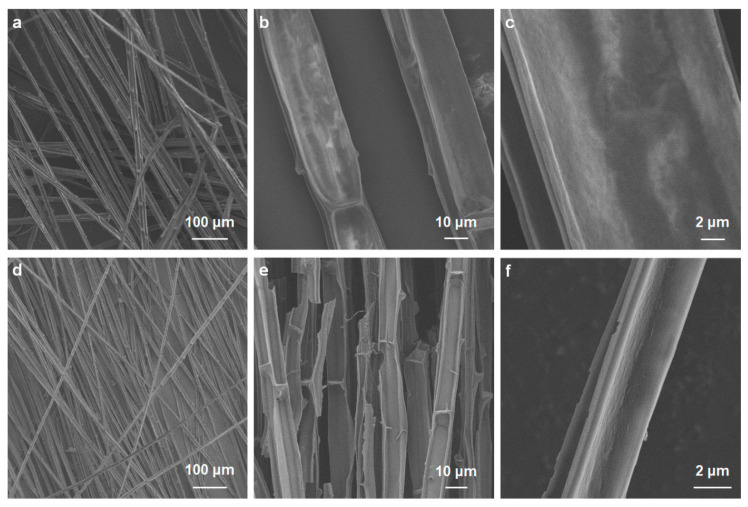
SEM images of (**a**–**c**) raw cattail and (**d**–**f**) carbonized cattail (CC).

The carbonized cattail–agarose hydrogel (CCAH) membrane was prepared via a facile route (Figure 1). First, agarose hydrogel was produced through dissolving, freezing, and centrifugation. Next, cattail was calcined in an environment with N_2_. Afterward, the six cut carbonized cattails were bonded together with adhesive (agarose hydrogel) to form a CCAH membrane, which can be used for solar interface evaporation (Figure 1). Raw cattail is mainly composed of numerous fibers, each with a distinct cross-sectional diameter and a smooth longitudinal surface. The fiber’s interior is divided into numerous small regions by numerous nodes that resemble bamboo nodes (Figure 2a–c). The carbonized cattail was structurally identical to the original, but it was black in color with a smaller volume. The characteristic bamboo knot-like structure and smooth longitudinal surface were still present in the carbonized fiber, but its cross-sectional diameter was only around one-fifth that of the original fiber (Figure 2d–f). Carbonized fibers with a hollow, bamboo-like structure can be employed as micron-sized pipelines to transfer water because of the capillary phenomena of plants. Due to the abundance of water-transporting fibers in the CCAH membrane, it is anticipated that it will accomplish effective solar interface evaporation.

**Figure 3:**
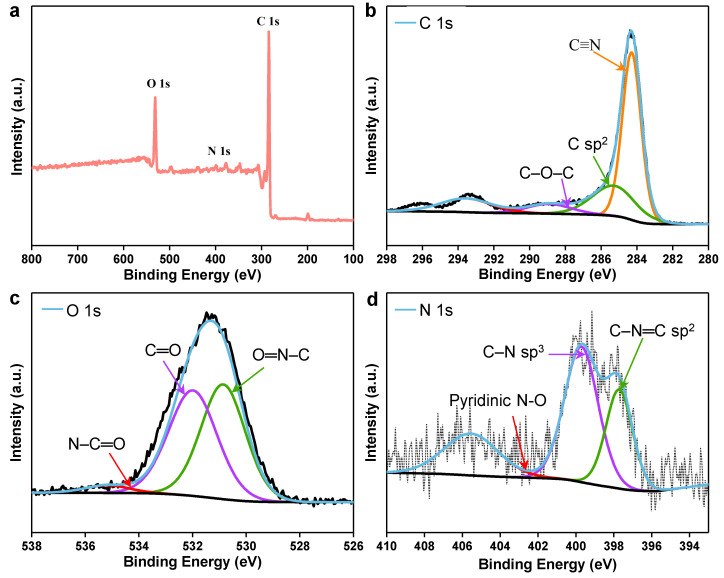
(**a**) Full XPS spectrum of CC. (**b**–**d**) High-resolution XPS spectra of (**b**) C 1s, (**c**) O 1s, (**d**) N 1s for CC.

The chemical makeup of unprocessed cattail and CC was investigated using the Fourier transform infrared spectra (FT-IR). The stretching vibrations of the O-H, CH_2_, and six-membered ring ether bonds are represented by the absorption peaks near 3385, 2920, and 1044 cm^−1^ in the FTIR spectra of raw cattail, respectively (Figure S1). In addition, the aldehyde, carboxyl, ketone, carbonyl, and ester groups were primarily responsible for the absorption of distinctive peaks at 1735, 1609, and 1516 cm^−1^ (Figure S1). The majority of the O-H and C-O groups were still present in CC after the carbonization process, ensuring high hydrophilicity of CC (Figure S1). The adsorption capability of the CCAH membrane was improved by these functional groups, which is advantageous for water filtration. Additionally, XPS was used to gather data on the surface chemistry of CC (Figure 3). The spectra demonstrate that CC comprises the elements C, O, and N (Figure 3a–d). A strong C-N/N-H peak at 399.9 eV in the N 1s spectrum confirms the presence of nitrogen functional groups in CC (Figure 3d). After carbonization, the remaining nitrogen functional groups can help the CCAH membrane become more hydrophilic. 

**Figure 4:**
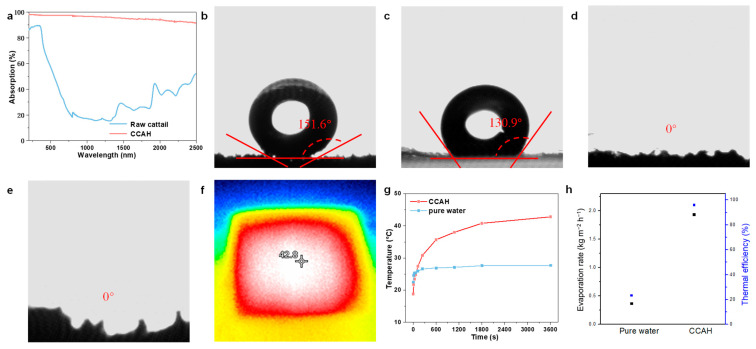
(**a**) The absorptions of raw cattail and CCAH membrane. (**b**,**c**) Digital images and contact angles of (**b**) top-view and (**c**) cross-sectional raw cattail. (**d**,**e**) Digital images and contact angles of (**d**) top-view and (**e**) cross-sectional CCAH membrane. (**f**) Infrared thermal image of CCAH membrane after one hour of irradiation (1 sun). (**g**) The temperature on the top surfaces of pure water and CCAH membrane under 1 sun irradiation versus time. (**h**) Evaporation rates and efficiency of pure water and CCAH membrane under 1 sun irradiation.

For the solar interface evaporation process, the optical property is just as critical as the structural characteristics and chemical components. Therefore, ultraviolet-visible near-infrared (UV-vis-NIR) absorption spectra (200 to 2500 nm) of raw cattail and CCAH membrane were measured. CCAH membrane absorbs around 97.4% of UV-visible light (280–780 nm) and about 90.1% of NIR light (780–2500 nm), both of which are much more than what raw cattail absorbs (Figure 4a). The above results demonstrate that the CCAH membrane exhibits strong absorption over the whole solar spectrum. In addition, investigations with contact angles were carried out to evaluate the wettability of materials. While raw cattail has a highly hydrophobic surface and cross-section resulting from its surface tension and microstructures (Figure 4b,c), the hollow structure and hydrophilic groups after carbonization, in contrast, give the CCAH membrane significant hydrophilicity (Figure 4d,e). 

In the water evaporation experiment, the CCAH membrane floating on the water surface demonstrated a faster heating rate and higher temperature compared to the pure water surface under one sun irradiation. The temperature of the pure water surface grew from 22.5 °C to 26.9 °C after 1 h of solar exposure (Figure 4g), but in the same situation, the temperature jumped quickly from 18.8 °C to 42.8 °C in the presence of the CCAH membrane (Figure 4f,g and Figure S2). Figure S2 demonstrates the quick temperature rise on the CCAH membrane, which reached 27.4 °C in 2 min and 40.8 °C within 30 min. Pure water evaporates at a rate of 0.37 kg m^−2^ h^−1^, but pure water with the CCAH membrane can evaporate at a rate of up to 1.93 kg m^−2^ h^−1^ (Figure 4h). The CCAH membrane has a higher evaporation rate than the majority of biochar solar interface evaporation devices reported so far (Table S1), at roughly 5.2 times faster than that of pure water. The evaporation efficiency of the CCAH membrane is calculated to be as high as 95.8% (Figure 4h), which is mostly attributable to its superior light absorption capacity and the potent capillary force effect brought on by its abundant hollow fibers.

**Figure 5:**
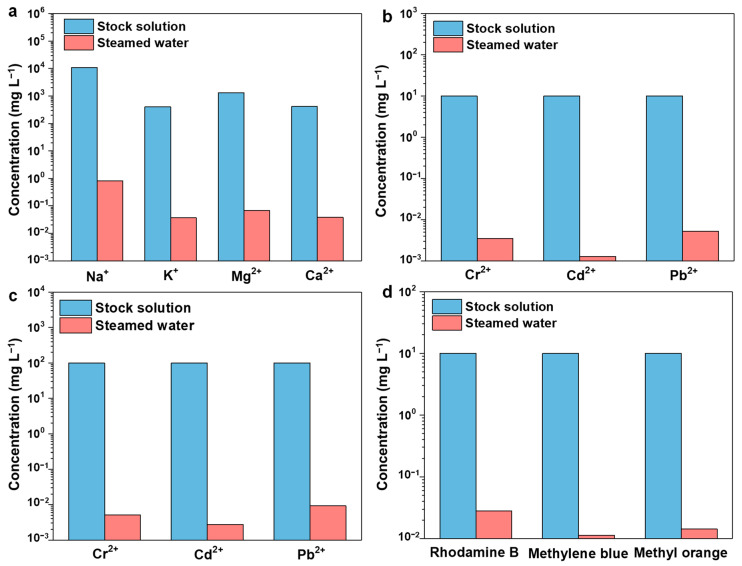
(**a**) The concentrations of simulated seawater before and after purification. (**b**,**c**) The concentrations of heavy metal ions (**b**): 10 ppm, (**c**): 100 ppm before and after purification. (**d**) The concentrations of dye solution before and after purification.

Under one sun exposure, the CCAH membrane was used to perform solar evaporation on simulated seawater that contains four ions (Na^+^, K^+^, Mg^2+^, and Ca^2+^). The concentrations of four ions in simulated seawater and desalination-produced steam water were measured using ICP optical emission spectroscopy (ICP-OES). The results revealed that the amounts of Na ^+^, Mg^2 +^, K ^+^, and Ca^2 +^ in the stock solution were 10,780 mg L^−1^, 1298 mg L^−1^, 400 mg L^−1^, and 410 mg L^−1^, respectively, whereas the amounts in the steamed water were 0.826 mg L^−1^, 0.0368 mg L^−1^, 0.0672 mg L^−1^, and 0.0379 mg L^−1^, respectively (Figure 5a, Table S2). The levels of four ions in steamed water were much lower than those recommended by the World Health Organization for drinking water [51]. The elimination rate of each ion was above 99.99% (Table S2). As shown in Table S3, the concentrations of these four ions in the steamed water obtained from CCAH are one or two orders of magnitude lower than those found in the majority of published works [37]. Moreover, the CCAH membrane has a better capacity to remove sodium ions as compared to an evaporator with high material cost and good overall performance documented in the literature [52]. Furthermore, no discernible buildup of salt or dirt on the CCAH membrane was observed, and the evaporation rate revealed no obvious change through multiple cycles (12 h per day, 5 days), which can be ascribed to gap dissolution and diffusion [53]. Specifically, the salt-blocking mechanism can be categorized as natural dissolution, with the salt dissolved through a water pump in the dark [54].

Different types of wastewater were generated with heavy metals or organic dyes to test the efficacy of CCAH membrane treatment. In the heavy metal (Pb^2+^, Cd^2+^, and Cr^2+^) wastewater purification experiment, wastewater was evaporated by one sun irradiation, and the resulting steamed water was collected. The concentrations of Cr^2+^ in sample 1, Cd^2+^ in sample 2, and Pb^2+^ in sample 3 were all 10 mg L^−1^. The concentration of Cr^2+^ in the steamed water of sample 1 was 3.43 μg L^−1^, that of Cd^2+^ in the steamed water of sample 2 was 1.72 μg L^−1^, and that of Pb^2+^ in the steamed water of sample 3 was 5.24 μg L^−1^, all as determined by ICP-OES measurement (Figure 5b, Table S4). The concentrations of Cr^2+^ in sample 4, Cd^2+^ in sample 5, and Pb^2+^ in sample 6 were all 100 mg L^−1^. The concentration of Cr^2+^ in the steamed water from sample 4 was 5.12 μg L^−1^, that of Cd^2+^ in the steamed water from sample 5 was 2.74 μg L^−1^, and that of Pb^2+^ in the steamed water from sample 6 was 9.17 μg L^−1^, all as determined by ICP measurements (Figure 5c, Table S5). The levels of heavy metal ions in steamed water from low (10 mg L^−1^)- and high (100 mg L^−1^)-concentration wastewater were reduced by four and five orders of magnitude, respectively (Figure 5b,c, Tables S4 and S5) and were below the WHO-recommended standard concentration [43,51]. The rejection rates of Cr^2+^, Cd^2+^, and Pb^2+^ for wastewater with low concentration (10 mg L^−1^) were 99.97%, 99.98%, and 99.95% (Table S4), respectively, whereas for wastewater with high concentration (100 mg L^−1^), the rejection rates were all higher than 99.99% (Table S5). Unexpectedly, the rejection rate of high-concentration ions was higher than that of low-concentration ions. Therefore, as a key advance in solar interface evaporation technology, we first propose that the biochar solar interface evaporation device (CCAH membrane) can be particularly successful in treating both low (10 mg L^−1^)- and high (100 mg L^−1^)-concentration heavy metal wastewater.

**Figure 6:**
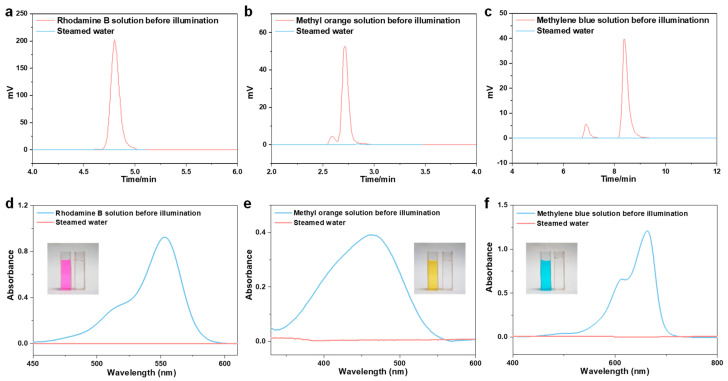
(**a**–**c**) The high-performance liquid chromatographic (HPLC) spectra of (**a**) Rhodamine B, (**b**) methyl orange, and (**c**) methylene blue solutions before and after purification. (**d**–**f**) UV-vis absorption spectroscopy of dye solutions (**d**) Rhodamine B, (**e**) methyl orange, and (**f**) methylene blue. Inset: The photographs of contaminated water (**d**): Rhodamine B, (**e**): methyl orange, and (**f**): methyl blue before and after solar purification.

To prepare the simulated dye wastewater (10 mg L^−1^), three typical organic dyes (Rhodamine B, methyl orange, and methyl blue) were utilized. The experimental process of water purification was the same as it is for wastewater containing heavy metals. The dye concentrations in the stock solution and the steamed water were determined using high-performance liquid chromatography (HPLC) and ultraviolet-visible spectrophotometry (UV-vis) (Figure 5d, Figure 6). Rhodamine B, methyl orange, and methyl blue concentrations in steamed water were decreased to 0.028 mg L^−1^, 0.011 mg L^−1^, and 0.014 mg L^−1^ (Figure 5d, Figure 6, and Table S6), with rejection rates calculated to be 99.72%, 99.89%, and 99.86% (Table S6), respectively. Additionally, the steamed water turned clear and translucent when compared to the colored stock solution (inset pictures in Figure 6d–f). Overall, the CCAH membrane exhibited great water purification capabilities and rejected the majority of salts, heavy metal ions, and organic dyes in the aqueous solution.

## 4. Conclusions

In conclusion, based on a carbonized cattail and agarose hydrogel (CCAH), we successfully created a simple, efficient, low-cost, and environmentally friendly bio-based solar interfacial evaporation device. The CCAH membrane is able to successfully perform photothermal conversion and evaporate water because of its abundance of microchannels that resemble bamboo knots, superior hydrophilicity, and light absorption capacity. The water evaporation rate and efficiency under one sun irradiation were 1.93 kg m^−2^ h^−1^ and 95.8%, respectively. The evaporation rate of the CCAH membrane was roughly 5.2 times that of pure water. More crucially, salts, heavy metals, and dyes were almost entirely removed from the steam water when the CCAH membrane was used in solar-driven water treatment. This discovery opens up new and extensive opportunities for saltwater desalination and wastewater purification because of the excellent water treatment comprehensive capabilities of the CCAH membrane.

## Data Availability

The data presented in this study are available on request from the corresponding author.

## Supplementary Materials

The following supporting information can be downloaded at: https://www.mdpi.com/article/10.3390/membranes12111076/s1, Figure S1: FT-IR spectra of raw cattail and CC; Figure S2: Time-dependent infrared images of pure water with CCAH membrane under 1 sun illumination; Figure S3: Mass change of simulated seawater with light irradiation (1 sun) of each cycle; Table S1: Comparison the performance of solar evaporator over different biochar materials; Table S2: The ICP results of simulated seawater before and after solar purification with CCAH membrane; Table S3: Comparison of ion concentration in steamed water produced by evaporators of different materials; Table S4: The ICP results of polluted water containing heavy metal ions before (c=10 ppm) and after solar purification with CCAH membrane; Table S5: The ICP results of polluted water containing heavy metal ions before (c = 100 ppm) and after solar purification with CCAH membrane; Table S6: The HPLC results of water samples containing dyes before and after solar purification with CCAH membrane. References [27,35,45,46,48] are cited in the supplementary materials
