# Supplementary material for: A Simple and Efficient Solar Interfacial Evaporation Device Based on Carbonized Cattail and Agarose Hydrogel for Water Evaporation and Purification
Source: Membranes (Basel). 2022 Oct 30;12(11):1076. doi: 10.3390/membranes12111076 (PMC9692801; doi:10.3390/membranes12111076)
Supplement: Supplementary file 1 [file membranes-12-01076-s001.zip › membranes-1948004-supplementary.pdf]

Supplementary Material

A Simple and Efficient Solar Interfacial Evaporation Device based on Carbonized Cattail and Agarose Hydrogel for Water Evaporation and Purification

Liang Wang [1], Jilei Wei [2], Chen Zhou [2,3,*], Shengyang Yang [2,*]

[1] School of Chemistry and Materials Engineering, Nanjing Polytechnic Institute, 188 Xinle Road, Nanjing 210048, China
[2] Department of Chemistry and Chemical Engineering, Yangzhou University, 180 Siwangting Road, Yangzhou 225002, China
[3] Department of Physical Sciences, University of Central Missouri, Warrensburg, MO 64093, USA
* Correspondence: zhou@ucmo.edu (C.Z.); syyang@yzu.edu.cn (S.Y.)

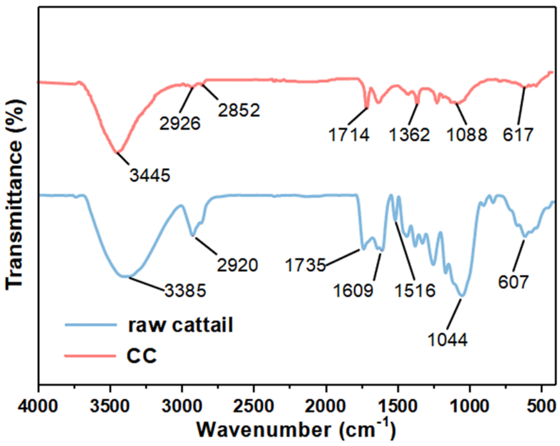

Figure S1. FT-IR spectra of raw cattail and CC.

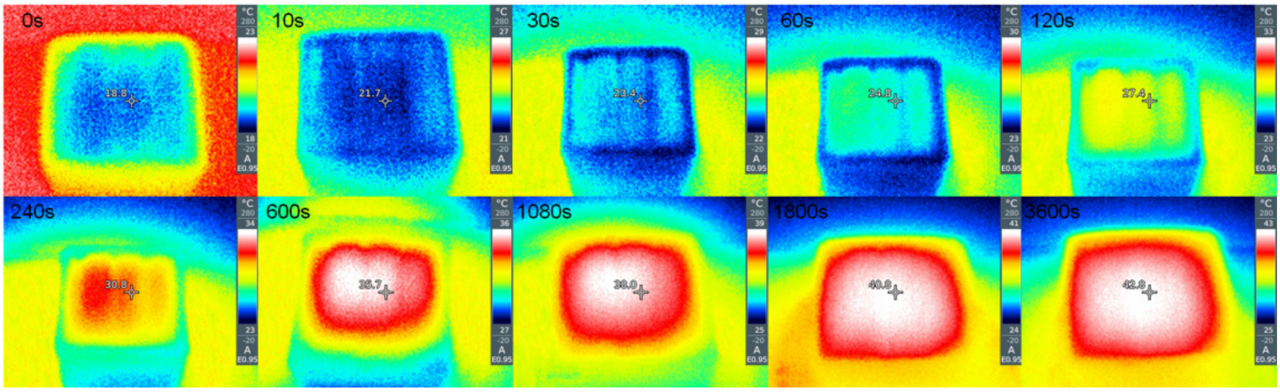

Figure S2. Time-dependent infrared images of pure water with CCAH membrane under 1 sun illumination.

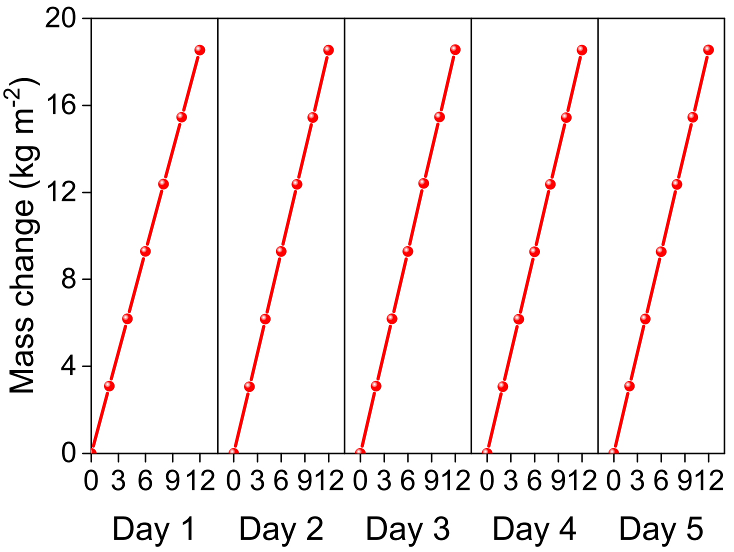

Figure S3. Mass change of simulated seawater with light irradiation (1 sun) of each cycle.

Table S1. Comparison the performance of solar evaporator over different biochar materials.

Evaporator	Light Intensity ($kW\ m^{-2}$)	Rate ($kg\ m^{-2}\ h^{-1}$)	Efficiency (%)	References
carbonized mushroom	1	1.475	78	[1]
CL-CNF (carbonized loofah)	1	1.72	92.5	[2]
Surface-Carbonized Bamboo	1	1.67	95.2	[3]
carbonized E. prolifera	1	1.30	84	[4]
CCAH (carbonized cattail)	1	1.93	95.8	Our work

Table S2. The ICP results of simulated seawater before and after solar purification with CCAH membrane.

Ions	Stock Solution (mg L^{-1})	Steamed Water (mg L^{-1})	Rejection Eficiency (%)
Na^+	10780	0.826	99.99
Mg^{2+}	1298	0.0368	99.99
K^+	400	0.0672	99.99
Ca^{2+}	410	0.0379	99.99

Table S3. Comparison of ion concentration in steamed water produced by evaporators of different materials

Evaporator	Na^+ (mg L^{-1})	Mg^{2+} (mg L^{-1})	K^+ (mg L^{-1})	Ca^{2+} (mg L^{-1})	References
Ag/PPy-PMBA-BrILs	~2	~0.5	~0.8	~1	[5]
HPH-based SVG	~6	0.95	~0.9	~0.9	[6]
rGOHM	~1	~0.3	~0.15	~0.4	[7]
SCG	~1.2	~0.15	~0.15	~0.8	[8]
CBS-Ti_3C_2	~5	~0.2	~0.8	~0.5	[9]
HHEs	~1.2	~0.2	~0.5	~0.4	[10]
SSC5	13.5	1.62	4.47	1.88	[11]
CCAH	0.826	0.0368	0.0672	0.0379	Our work

Table S4. The ICP results of polluted water containing heavy metal ions before (c=10 ppm) and after solar purification with CCAH membrane.

Sample Number	Ions	Stock Solution ($mg\ L^{-1}$)	Steamed Water ($\mu g\ L^{-1}$)	Rejection Eficiency (%)
Sample 1	Cr^{2+}	10	3.43	99.97
Sample 2	Cd^{2+}	10	1.72	99.98
Sample 3	Pb^{2+}	10	5.24	99.95

Table S5. The ICP results of polluted water containing heavy metal ions before (c=100 ppm) and after solar purification with CCAH membrane.

Sample Number	Ions	Stock Solution ($mg\ L^{-1}$)	Steamed Water ($\mu g\ L^{-1}$)	Rejection Eficiency (%)
Sample 4	Cr^{2+}	100	5.12	99.99
Sample 5	Cd^{2+}	100	2.74	99.99
Sample 6	Pb^{2+}	100	9.17	99.99

Table S6. The HPLC results of water samples containing dyes before and after solar purification with CCAH membrane.

Dyes	Stock Solution ($mg\ L^{-1}$)	Steamed Water ($mg\ L^{-1}$)	Rejection Eficiency (%)
Rhodamine B	10	0.028	99.72

Methyl orange	10	0.011	99.89
Methylene blue	10	0.014	99.86

References

[1] N. Xu, X. Hu, W. Xu, X. Li, L. Zhou, S. Zhu, and J. Zhu, Mushrooms as efficient solar steam-generation devices, Adv. Mater., 2017, No. 1606762.

[2] C. Zhang, B. Yuan,Y. Liang, L.Yang, H. Chen, Carbon nanofibers enhanced solar steam generation device based on loofah biomass for water purification, Materials Chemistry and Physics, 2021, 258, No. 123998.

[3] J. Liu, J. Yao, Y. Yuan, Q. Liu, W. Zhang, X. Zhang, J. Gu, Surface-carbonized bamboos with multilevel functional biostructures deliver high photothermal water evaporation performance, Adv. Sustain. Syst., 2020, No. 2000126.

[4] L. Yang, G. Chen, N. Zhang, Y. Xu, X. Xu, Sustainable biochar-based solar absorbers for high-performance solar-driven steam generation and water purification, ACS Sustainable Chem. Eng., 2019, 7: 19311−19320.

[5] C. Xiao, W. Liang, Q. Hasi, L. Chen, J. He, F. Liu, C. Wang, H. Sun, Z. Zhu, A. Li, Ag/polypyrrole co-modified poly(ionic liquid)s hydrogels as efficient solar generators for desalination. Materials Today Energy, 2020, 16, No. 100417.

[6] F. Zhu, L.Wang, B. Demir, M. An, Z. Wu, J. Yin, R. Xiao, Q. Zheng, J. Qian, Accelerating solar desalination in brine through ion activated hierarchically porous polyion complex hydrogels, Mater. Horiz., 2020, 7: 3187-3195.

[7] P. Zhuang, D. Li, N. Xu, X. Yu, L. Zhou, Stable self-floating reduced graphene oxide hydrogel membrane for high rate of solar vapor evaporation under 1 sun, Global Challenges, 2021, 5, No. 2000053.

[8] L. Zhao, C. Du,C. Zhou, S. Sun, Y. Jia, J. Yuan, G. Song, X. Zhou, Q. Zhao, S. Yang.Structurally ordered AgNPs@C_3N_4/GO membranes toward solar-driven freshwater generation, ACS Sustainable Chem. Eng., 2020, 8: 4362−4370.

[9] Z. Wang, K. Yu, S. Gong, H. Mao, R. Huang, Z. Zhu. Cu_3BiS_3 / MXenes with excellent solar−thermal conversion for continuous and efficient seawater desalination, ACS Appl. Mater. Interfaces, 2021, 13: 16246−16258.

[10] Y. Guo, H. Lu, F. Zhao, X. Zhou, W. Shi, G. Yu. Biomass-derived hybrid hydrogel evaporators for cost-effective solar water purification, Adv. Mater., 2020, 32, No. 1907061.

[11] J. Yin, J. Xu, W. Xu, S. Liu, W. Li, Z. Fang, C. Lu, Z. Xu, Tuning the wettability of solar absorbers towards high-efficiency solar vapor generation, Applied Thermal Engineering, 2021, 183, No. 116224.
